# MARITAL STATUS AND QUALITY OF END-OF-LIFE EXPERIENCES AMONG OLDER US ADULTS

**DOI:** 10.1093/geroni/igae098.2027

**Published:** 2024-12-31

**Authors:** Kafayat Mahmoud, Deborah Carr, Jarron Saint Onge

**Affiliations:** Boston University, Boston, Massachusetts, United States; Boston University, Boston, Massachusetts, United States; University of Kansas, Lawrence, Kansas, United States

## Abstract

Understanding the quality of end-of-life experiences is a critical goal, because many older adults experience discomfort or care discordant with their preferences at the end of life. While extensive research documents the protective effects of marriage for health, we know of no studies focusing specifically on marital status differences in end-of-life well-being and care. We examine the extent to which marital status (married, remarried, divorced, widowed, never married) affects end-of-life physical and emotional well-being, quality of care, and dignified care, and whether these associations differ by gender. Using 13 waves of data from the National Health and Aging Trends Study (NHATS), we estimate multivariable multinomial and binomial logistic regression models to evaluate how marital statuses affect diverse indicators of end-of-life well-being and gender differences therein. Our analyses show that married persons do not fare uniformly better than their unmarried counterparts; rather, patterns differ across outcomes. Widowed and remarried persons have a higher likelihood of reporting excellent or very good care at the end of life, relative to married persons. Widowed older adults also are more likely to report receiving care concordant with their preferences. Divorced older adults report poorer pain management, while remarried and never married older adults report less sadness relative to married persons. Patterns do not differ significantly by gender. Unmarried or remarried persons may perceive a more urgent need to do advance care planning, which can enhance the quality of dying experiences. We discuss how marital relationships more broadly shape end-of-life experiences.

